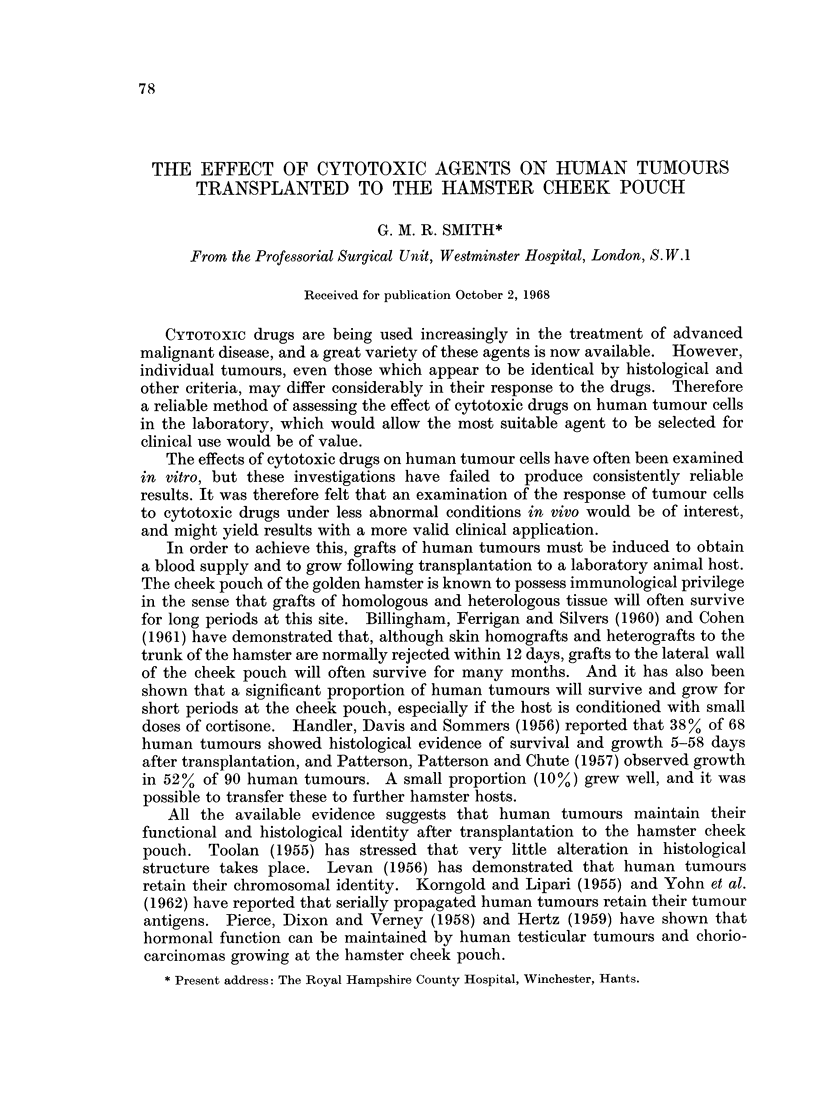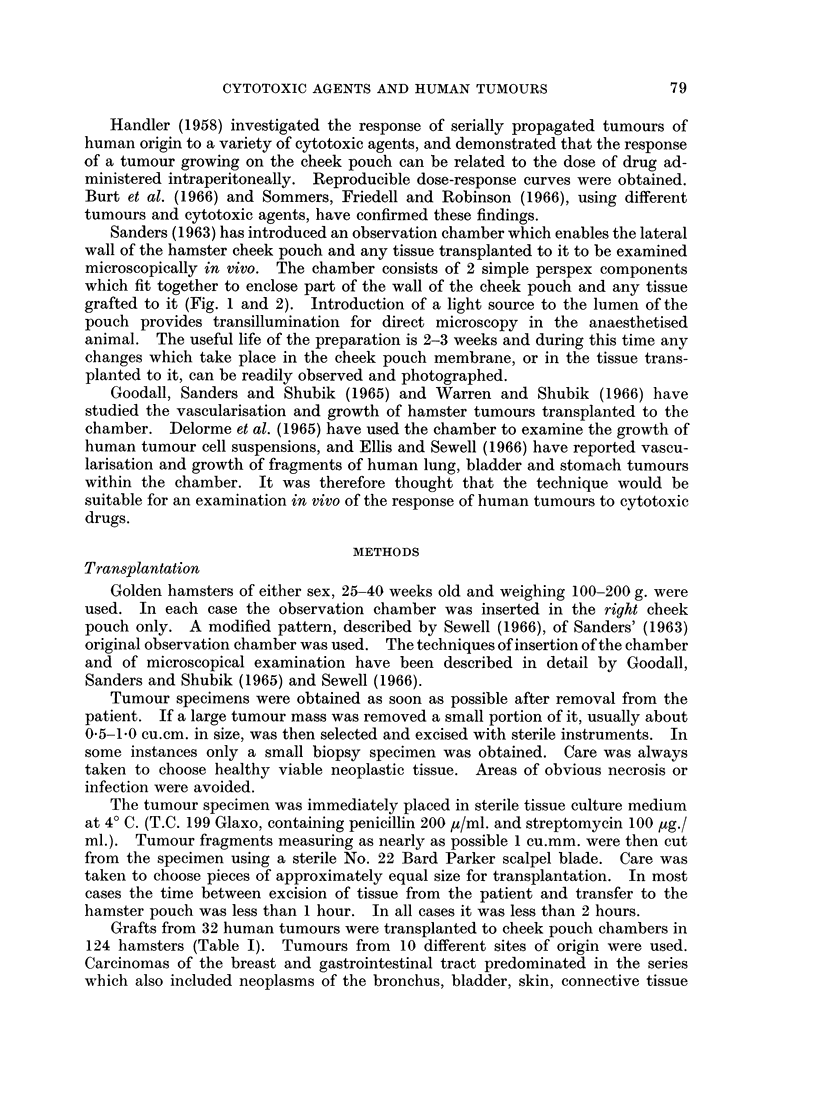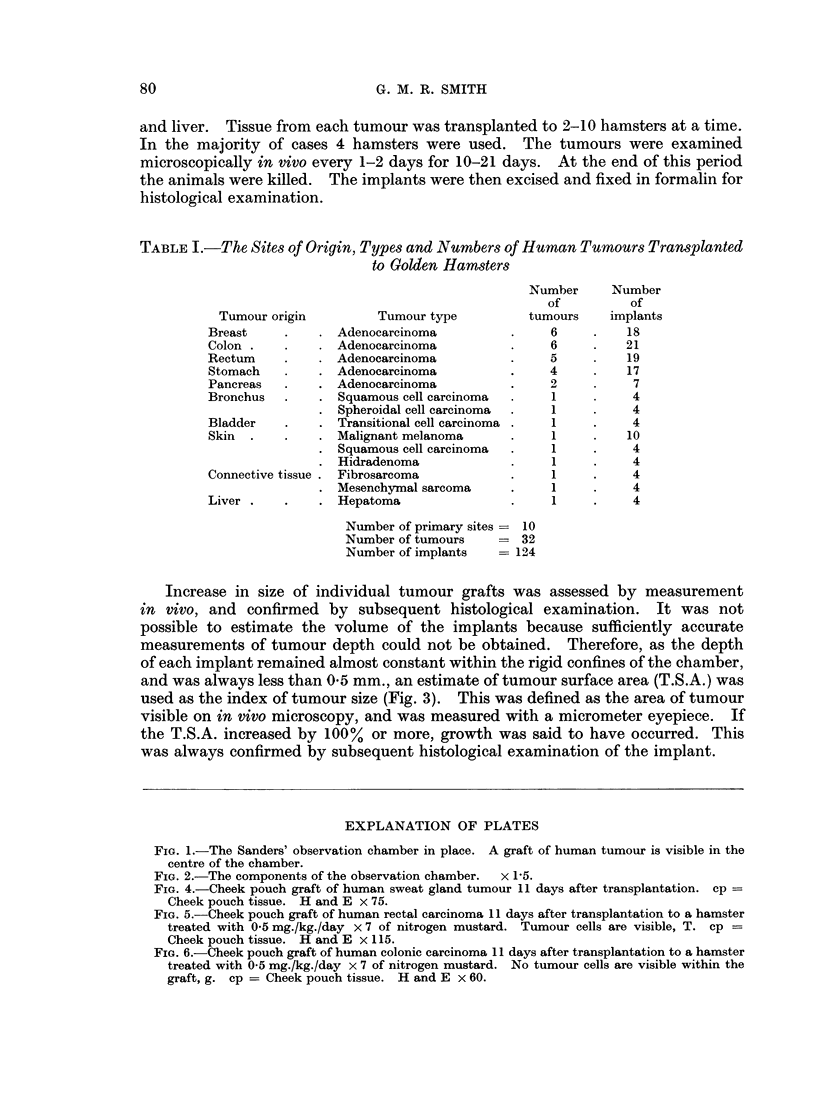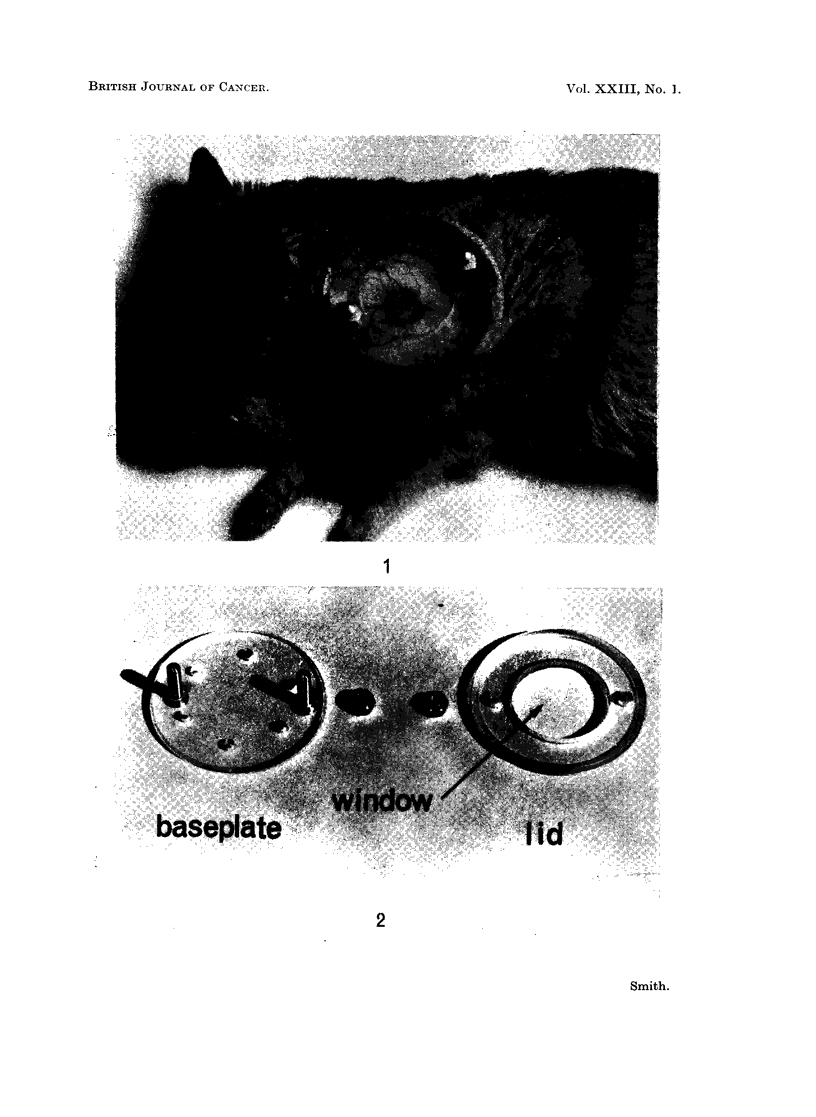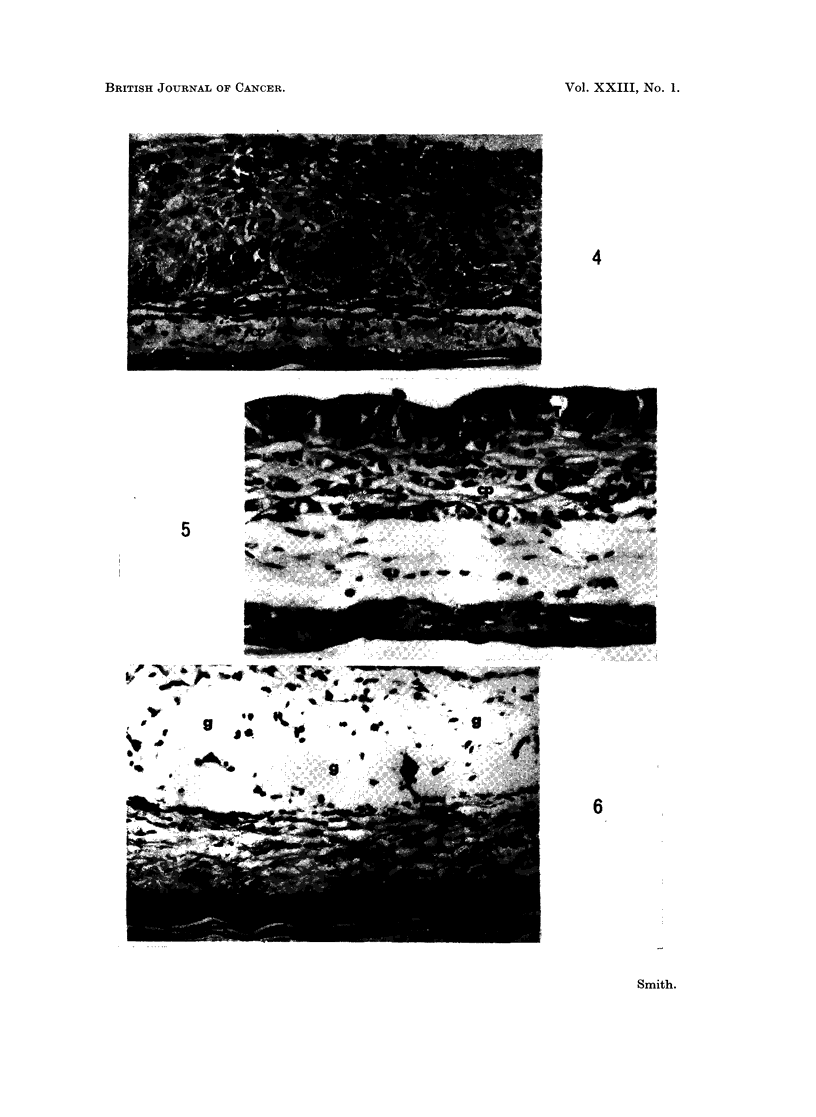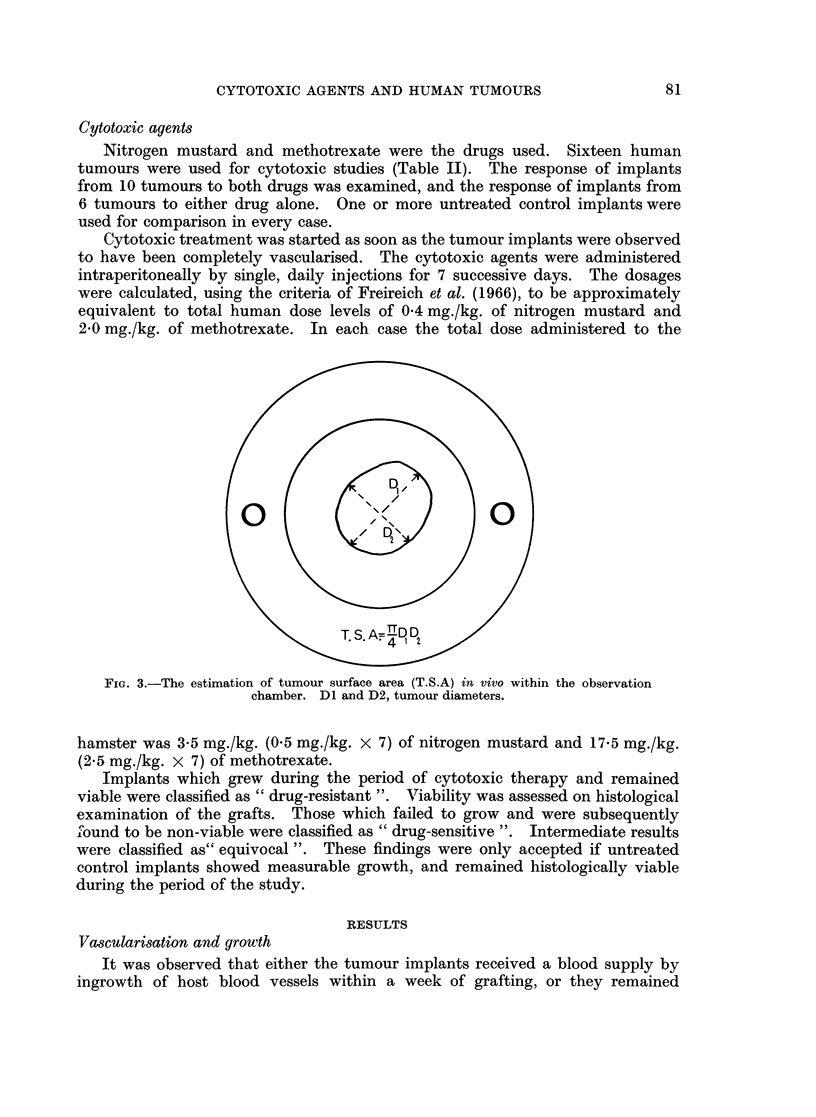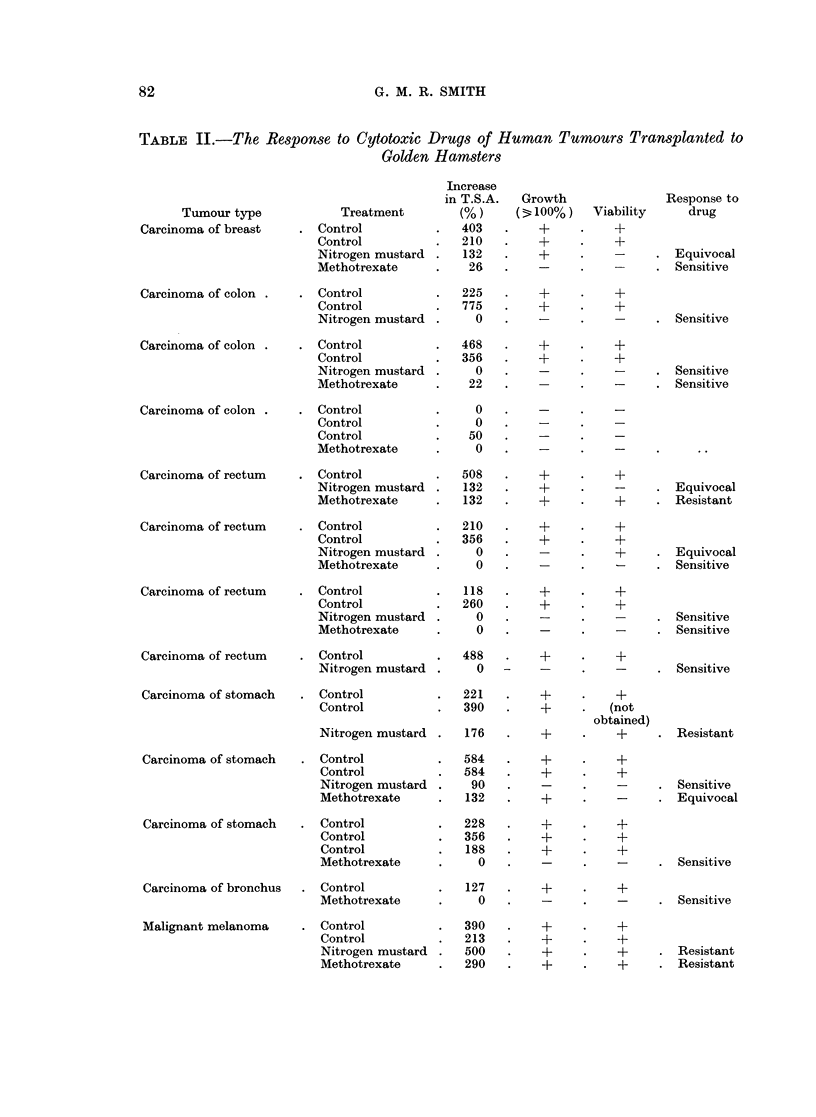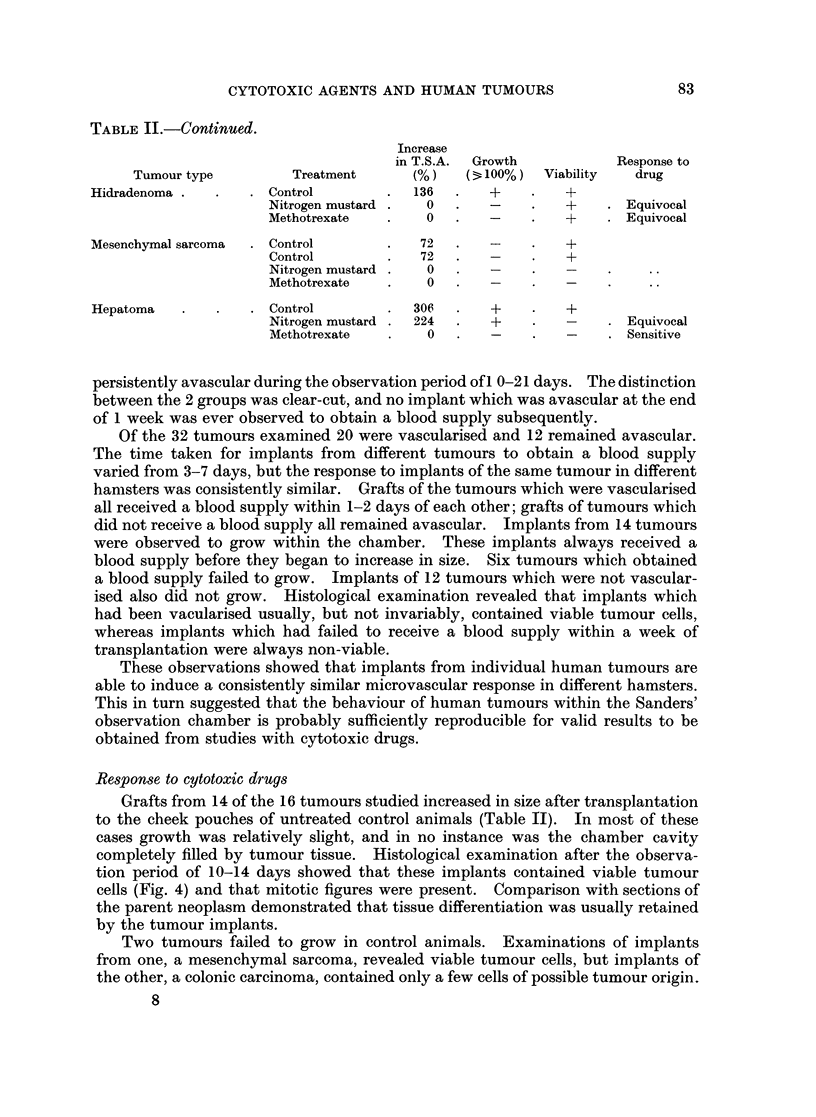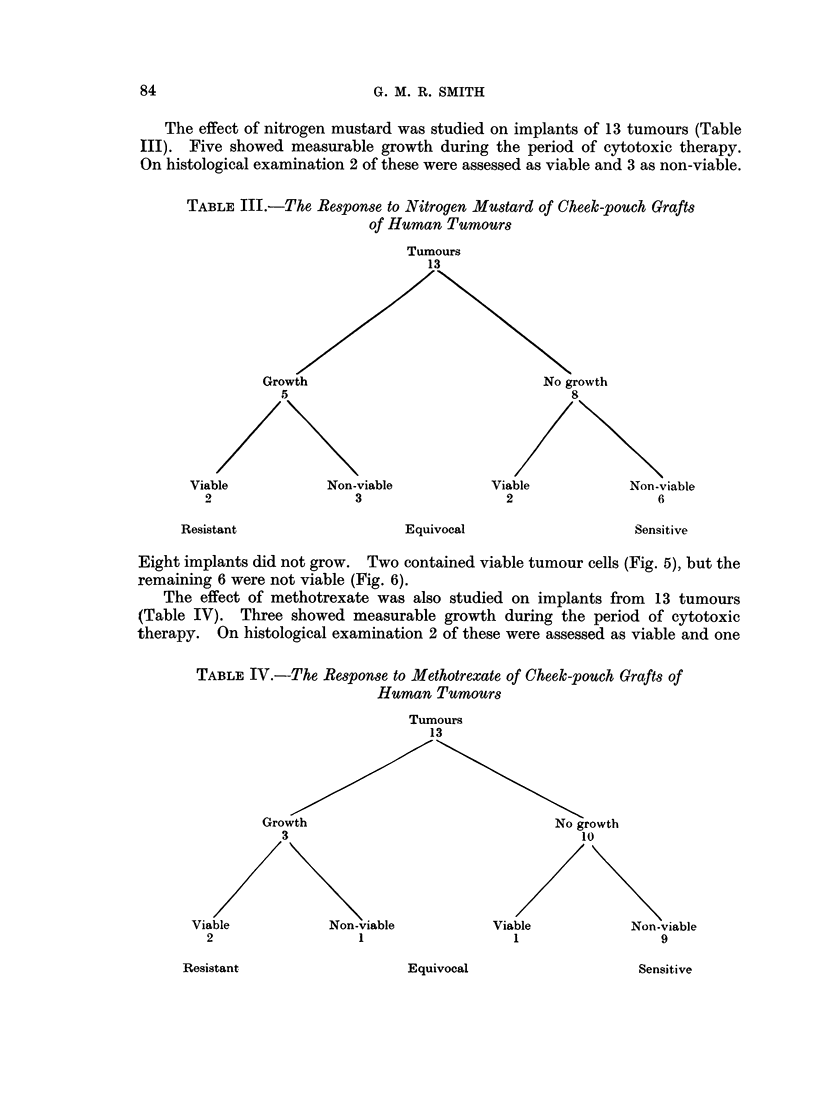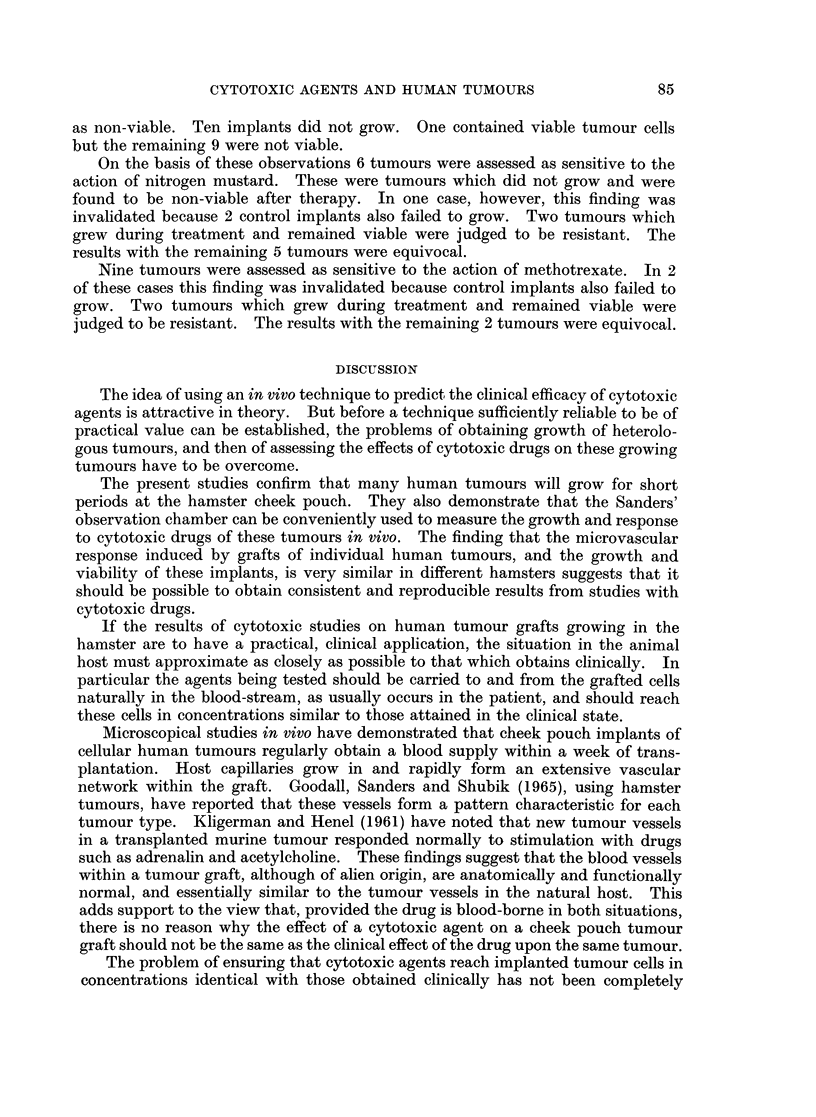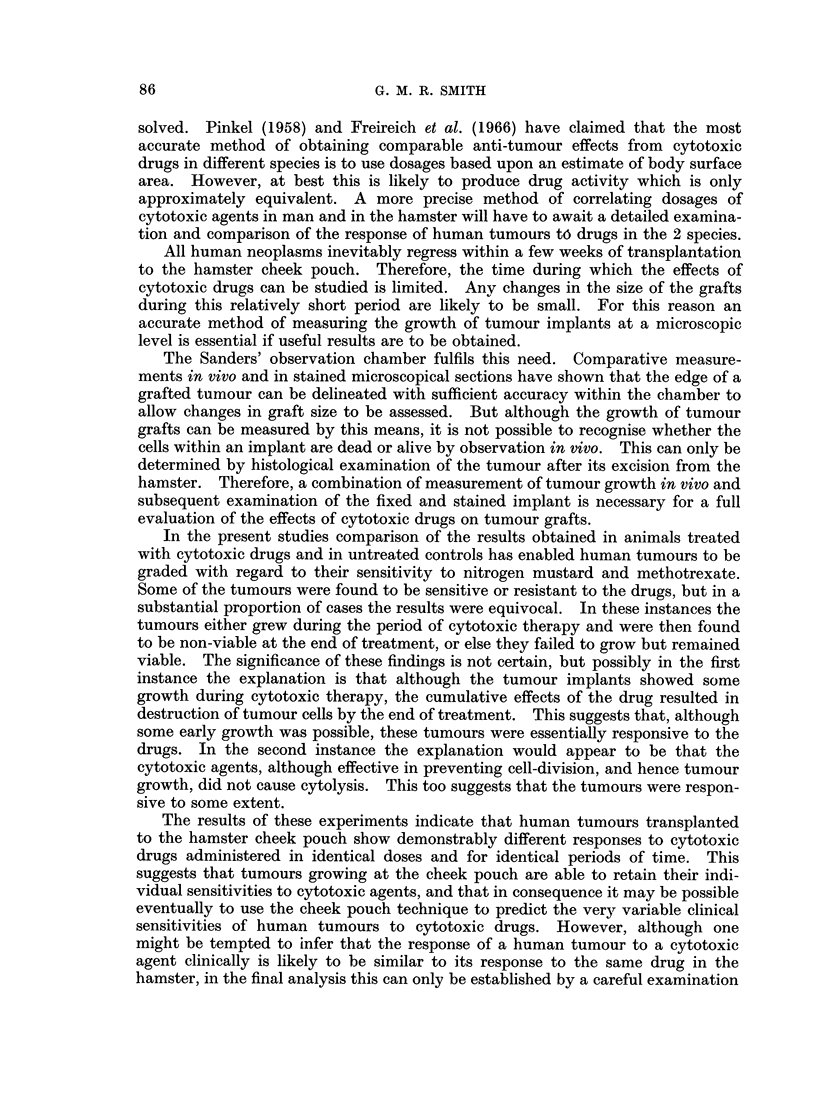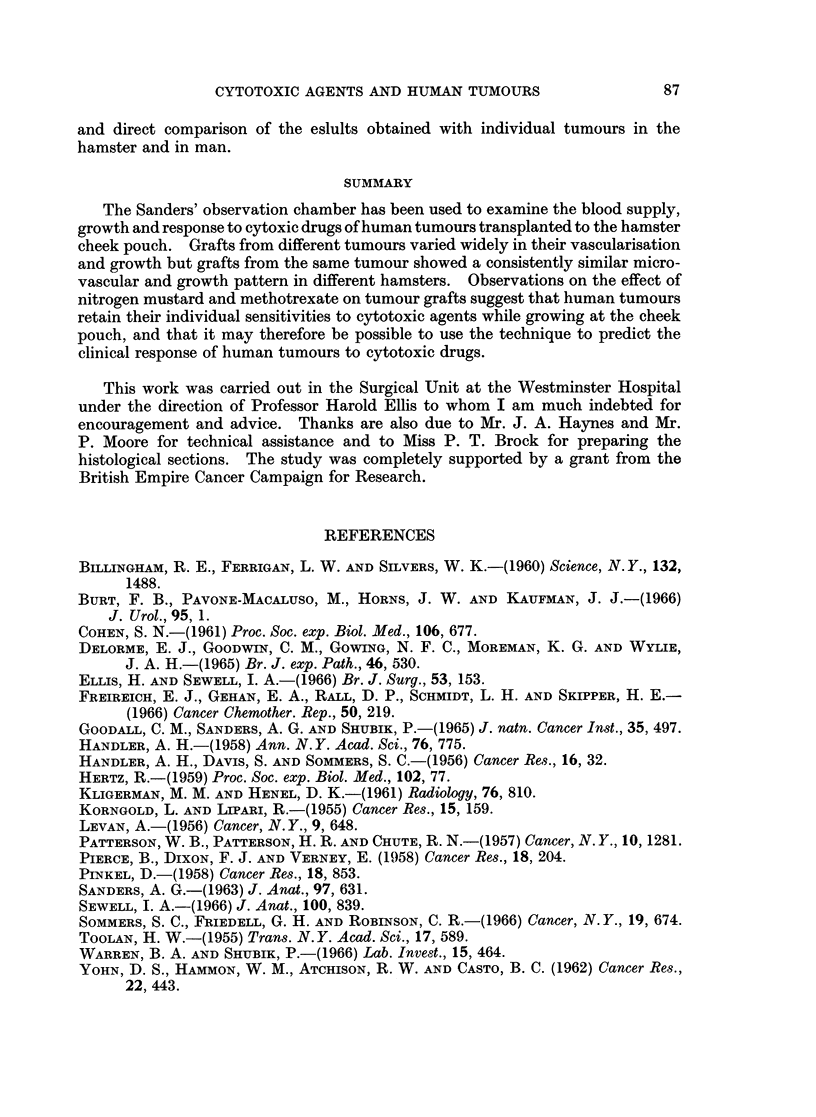# The effect of cytotoxic agents on human tumours transplanted to the hamster cheek pouch.

**DOI:** 10.1038/bjc.1969.13

**Published:** 1969-03

**Authors:** G. M. Smith

## Abstract

**Images:**


### THE EFFECT OF CYTOTOXIC AGENTS ON HUMAN TUMOURS TRANSPLANTED TO THE HAMSTER CHEEK POUCH

G. M. R. SMITH*

*From the Professorial Surgical Unit, Westminster Hospital, London, S.W.1*

Received for publication October 2, 1968

CYTOTOXIC drugs are being used increasingly in the treatment of advanced malignant disease, and a great variety of these agents is now available. However, individual tumours, even those which appear to be identical by histological and other criteria, may differ considerably in their response to the drugs. Therefore a reliable method of assessing the effect of cytotoxic drugs on human tumour cells in the laboratory, which would allow the most suitable agent to be selected for clinical use would be of value.

The effects of cytotoxic drugs on human tumour cells have often been examined *in vitro*, but these investigations have failed to produce consistently reliable results. It was therefore felt that an examination of the response of tumour cells to cytotoxic drugs under less abnormal conditions *in vivo* would be of interest, and might yield results with a more valid clinical application.

In order to achieve this, grafts of human tumours must be induced to obtain a blood supply and to grow following transplantation to a laboratory animal host. The cheek pouch of the golden hamster is known to possess immunological privilege in the sense that grafts of homologous and heterologous tissue will often survive for long periods at this site. Billingham, Ferrigan and Silvers (1960) and Cohen (1961) have demonstrated that, although skin homografts and heterografts to the trunk of the hamster are normally rejected within 12 days, grafts to the lateral wall of the cheek pouch will often survive for many months. And it has also been shown that a significant proportion of human tumours will survive and grow for short periods at the cheek pouch, especially if the host is conditioned with small doses of cortisone. Handler, Davis and Sommers (1956) reported that 38% of 68 human tumours showed histological evidence of survival and growth 5–58 days after transplantation, and Patterson, Patterson and Chute (1957) observed growth in 52% of 90 human tumours. A small proportion (10%) grew well, and it was possible to transfer these to further hamster hosts.

All the available evidence suggests that human tumours maintain their functional and histological identity after transplantation to the hamster cheek pouch. Toolan (1955) has stressed that very little alteration in histological structure takes place. Levan (1956) has demonstrated that human tumours retain their chromosomal identity. Korngold and Lipari (1955) and Yohn *et al.* (1962) have reported that serially propagated human tumours retain their tumour antigens. Pierce, Dixon and Verney (1958) and Hertz (1959) have shown that hormonal function can be maintained by human testicular tumours and choriocarcinomas growing at the hamster cheek pouch.

* Present address: The Royal Hampshire County Hospital, Winchester, Hants.

Handler (1958) investigated the response of serially propagated tumours of human origin to a variety of cytotoxic agents, and demonstrated that the response of a tumour growing on the cheek pouch can be related to the dose of drug administered intraperitoneally. Reproducible dose-response curves were obtained. Burt *et al.* (1966) and Sommers, Friedell and Robinson (1966), using different tumours and cytotoxic agents, have confirmed these findings.

Sanders (1963) has introduced an observation chamber which enables the lateral wall of the hamster cheek pouch and any tissue transplanted to it to be examined microscopically *in vivo.* The chamber consists of 2 simple perspex components which fit together to enclose part of the wall of the cheek pouch and any tissue grafted to it (Fig. 1 and 2). Introduction of a light source to the lumen of the pouch provides transillumination for direct microscopy in the anaesthetised animal. The useful life of the preparation is 2–3 weeks and during this time any changes which take place in the cheek pouch membrane, or in the tissue transplanted to it, can be readily observed and photographed.

Goodall, Sanders and Shubik (1965) and Warren and Shubik (1966) have studied the vascularisation and growth of hamster tumours transplanted to the chamber. Delorme *et al.* (1965) have used the chamber to examine the growth of human tumour cell suspensions, and Ellis and Sewell (1966) have reported vascularisation and growth of fragments of human lung, bladder and stomach tumours within the chamber. It was therefore thought that the technique would be suitable for an examination *in vivo* of the response of human tumours to cytotoxic drugs.

### METHODS

### *Transplantation*

Golden hamsters of either sex, 25–40 weeks old and weighing 100–200 g. were used. In each case the observation chamber was inserted in the *right* cheek pouch only. A modified pattern, described by Sewell (1966), of Sanders' (1963) original observation chamber was used. The techniques of insertion of the chamber and of microscopical examination have been described in detail by Goodall, Sanders and Shubik (1965) and Sewell (1966).

Tumour specimens were obtained as soon as possible after removal from the patient. If a large tumour mass was removed a small portion of it, usually about 0·5–1·0 cu.cm. in size, was then selected and excised with sterile instruments. In some instances only a small biopsy specimen was obtained. Care was always taken to choose healthy viable neoplastic tissue. Areas of obvious necrosis or infection were avoided.

The tumour specimen was immediately placed in sterile tissue culture medium at 4° C. (T.C. 199 Glaxo, containing penicillin 200 $\mu$/ml. and streptomycin 100 $\mu$g./ml.). Tumour fragments measuring as nearly as possible 1 cu.mm. were then cut from the specimen using a sterile No. 22 Bard Parker scalpel blade. Care was taken to choose pieces of approximately equal size for transplantation. In most cases the time between excision of tissue from the patient and transfer to the hamster pouch was less than 1 hour. In all cases it was less than 2 hours.

Grafts from 32 human tumours were transplanted to cheek pouch chambers in 124 hamsters (Table I). Tumours from 10 different sites of origin were used. Carcinomas of the breast and gastrointestinal tract predominated in the series which also included neoplasms of the bronchus, bladder, skin, connective tissue and liver. Tissue from each tumour was transplanted to 2–10 hamsters at a time. In the majority of cases 4 hamsters were used. The tumours were examined microscopically *in vivo* every 1–2 days for 10–21 days. At the end of this period the animals were killed. The implants were then excised and fixed in formalin for histological examination.

TABLE I.—*The Sites of Origin, Types and Numbers of Human Tumours Transplanted to Golden Hamsters*

| Tumour origin | Tumour type | Number of tumours | Number of implants |
|---|---|---|---|
| Breast | Adenocarcinoma | 6 | 18 |
| Colon | Adenocarcinoma | 6 | 21 |
| Rectum | Adenocarcinoma | 5 | 19 |
| Stomach | Adenocarcinoma | 4 | 17 |
| Pancreas | Adenocarcinoma | 2 | 7 |
| Bronchus | Squamous cell carcinoma | 1 | 4 |
| | Spheroidal cell carcinoma | 1 | 4 |
| Bladder | Transitional cell carcinoma | 1 | 4 |
| Skin | Malignant melanoma | 1 | 10 |
| | Squamous cell carcinoma | 1 | 4 |
| | Hidradenoma | 1 | 4 |
| Connective tissue | Fibrosarcoma | 1 | 4 |
| | Mesenchymal sarcoma | 1 | 4 |
| Liver | Hepatoma | 1 | 4 |

Number of primary sites = 10
Number of tumours = 32
Number of implants = 124

Increase in size of individual tumour grafts was assessed by measurement *in vivo*, and confirmed by subsequent histological examination. It was not possible to estimate the volume of the implants because sufficiently accurate measurements of tumour depth could not be obtained. Therefore, as the depth of each implant remained almost constant within the rigid confines of the chamber, and was always less than 0·5 mm., an estimate of tumour surface area (T.S.A.) was used as the index of tumour size (Fig. 3). This was defined as the area of tumour visible on *in vivo* microscopy, and was measured with a micrometer eyepiece. If the T.S.A. increased by 100% or more, growth was said to have occurred. This was always confirmed by subsequent histological examination of the implant.

### EXPLANATION OF PLATES

FIG. 1.—The Sanders' observation chamber in place. A graft of human tumour is visible in the centre of the chamber.

FIG. 2.—The components of the observation chamber. × 1·5.

FIG. 4.—Cheek pouch graft of human sweat gland tumour 11 days after transplantation. cp = Cheek pouch tissue. H and E × 75.

FIG. 5.—Cheek pouch graft of human rectal carcinoma 11 days after transplantation to a hamster treated with 0·5 mg./kg./day × 7 of nitrogen mustard. Tumour cells are visible, T. cp = Cheek pouch tissue. H and E × 115.

FIG. 6.—Cheek pouch graft of human colonic carcinoma 11 days after transplantation to a hamster treated with 0·5 mg./kg./day × 7 of nitrogen mustard. No tumour cells are visible within the graft, g. cp = Cheek pouch tissue. H and E × 60.

1

2

Smith.

4

5

6

Smith.

*Cytotoxic agents*

Nitrogen mustard and methotrexate were the drugs used. Sixteen human tumours were used for cytotoxic studies (Table II). The response of implants from 10 tumours to both drugs was examined, and the response of implants from 6 tumours to either drug alone. One or more untreated control implants were used for comparison in every case.

Cytotoxic treatment was started as soon as the tumour implants were observed to have been completely vascularised. The cytotoxic agents were administered intraperitoneally by single, daily injections for 7 successive days. The dosages were calculated, using the criteria of Freireich *et al.* (1966), to be approximately equivalent to total human dose levels of 0·4 mg./kg. of nitrogen mustard and 2·0 mg./kg. of methotrexate. In each case the total dose administered to the

FIG. 3.—The estimation of tumour surface area (T.S.A) *in vivo* within the observation chamber. D1 and D2, tumour diameters.

hamster was 3·5 mg./kg. (0·5 mg./kg. × 7) of nitrogen mustard and 17·5 mg./kg. (2·5 mg./kg. × 7) of methotrexate.

Implants which grew during the period of cytotoxic therapy and remained viable were classified as " drug-resistant ". Viability was assessed on histological examination of the grafts. Those which failed to grow and were subsequently found to be non-viable were classified as " drug-sensitive ". Intermediate results were classified as" equivocal ". These findings were only accepted if untreated control implants showed measurable growth, and remained histologically viable during the period of the study.

### RESULTS

*Vascularisation and growth*

It was observed that either the tumour implants received a blood supply by ingrowth of host blood vessels within a week of grafting, or they remained

TABLE II.—*The Response to Cytotoxic Drugs of Human Tumours Transplanted to Golden Hamsters*

| Tumour type | Treatment | Increase in T.S.A. (%) | Growth (⩾100%) | Viability | Response to drug |
|---|---|---|---|---|---|
| Carcinoma of breast | Control | 403 | + | + | |
| | Control | 210 | + | + | |
| | Nitrogen mustard | 132 | + | — | Equivocal |
| | Methotrexate | 26 | — | — | Sensitive |
| Carcinoma of colon | Control | 225 | + | + | |
| | Control | 775 | + | + | |
| | Nitrogen mustard | 0 | — | — | Sensitive |
| Carcinoma of colon | Control | 468 | + | + | |
| | Control | 356 | + | + | |
| | Nitrogen mustard | 0 | — | — | Sensitive |
| | Methotrexate | 22 | — | — | Sensitive |
| Carcinoma of colon | Control | 0 | — | — | |
| | Control | 0 | — | — | |
| | Control | 50 | — | — | |
| | Methotrexate | 0 | — | — | .. |
| Carcinoma of rectum | Control | 508 | + | + | |
| | Nitrogen mustard | 132 | + | — | Equivocal |
| | Methotrexate | 132 | + | + | Resistant |
| Carcinoma of rectum | Control | 210 | + | + | |
| | Control | 356 | + | + | |
| | Nitrogen mustard | 0 | — | + | Equivocal |
| | Methotrexate | 0 | — | — | Sensitive |
| Carcinoma of rectum | Control | 118 | + | + | |
| | Control | 260 | + | + | |
| | Nitrogen mustard | 0 | — | — | Sensitive |
| | Methotrexate | 0 | — | — | Sensitive |
| Carcinoma of rectum | Control | 488 | + | + | |
| | Nitrogen mustard | 0 | — | — | Sensitive |
| Carcinoma of stomach | Control | 221 | + | + | |
| | Control | 390 | + | (not obtained) | |
| | Nitrogen mustard | 176 | + | + | Resistant |
| Carcinoma of stomach | Control | 584 | + | + | |
| | Control | 584 | + | + | |
| | Nitrogen mustard | 90 | — | — | Sensitive |
| | Methotrexate | 132 | + | — | Equivocal |
| Carcinoma of stomach | Control | 228 | + | + | |
| | Control | 356 | + | + | |
| | Control | 188 | + | + | |
| | Methotrexate | 0 | — | — | Sensitive |
| Carcinoma of bronchus | Control | 127 | + | + | |
| | Methotrexate | 0 | — | — | Sensitive |
| Malignant melanoma | Control | 390 | + | + | |
| | Control | 213 | + | + | |
| | Nitrogen mustard | 500 | + | + | Resistant |
| | Methotrexate | 290 | + | + | Resistant |

TABLE II.—*Continued.*

| Tumour type | Treatment | Increase in T.S.A. (%) | Growth (≥100%) | Viability | Response to drug |
|---|---|---|---|---|---|
| Hidradenoma | Control | 136 | + | + | |
| | Nitrogen mustard | 0 | − | + | Equivocal |
| | Methotrexate | 0 | − | + | Equivocal |
| Mesenchymal sarcoma | Control | 72 | − | + | |
| | Control | 72 | − | + | |
| | Nitrogen mustard | 0 | − | − | .. |
| | Methotrexate | 0 | − | − | .. |
| Hepatoma | Control | 306 | + | + | |
| | Nitrogen mustard | 224 | + | − | Equivocal |
| | Methotrexate | 0 | − | − | Sensitive |

persistently avascular during the observation period of1 0–21 days. The distinction between the 2 groups was clear-cut, and no implant which was avascular at the end of 1 week was ever observed to obtain a blood supply subsequently.

Of the 32 tumours examined 20 were vascularised and 12 remained avascular. The time taken for implants from different tumours to obtain a blood supply varied from 3–7 days, but the response to implants of the same tumour in different hamsters was consistently similar. Grafts of the tumours which were vascularised all received a blood supply within 1–2 days of each other; grafts of tumours which did not receive a blood supply all remained avascular. Implants from 14 tumours were observed to grow within the chamber. These implants always received a blood supply before they began to increase in size. Six tumours which obtained a blood supply failed to grow. Implants of 12 tumours which were not vascularised also did not grow. Histological examination revealed that implants which had been vacularised usually, but not invariably, contained viable tumour cells, whereas implants which had failed to receive a blood supply within a week of transplantation were always non-viable.

These observations showed that implants from individual human tumours are able to induce a consistently similar microvascular response in different hamsters. This in turn suggested that the behaviour of human tumours within the Sanders' observation chamber is probably sufficiently reproducible for valid results to be obtained from studies with cytotoxic drugs.

*Response to cytotoxic drugs*

Grafts from 14 of the 16 tumours studied increased in size after transplantation to the cheek pouches of untreated control animals (Table II). In most of these cases growth was relatively slight, and in no instance was the chamber cavity completely filled by tumour tissue. Histological examination after the observation period of 10–14 days showed that these implants contained viable tumour cells (Fig. 4) and that mitotic figures were present. Comparison with sections of the parent neoplasm demonstrated that tissue differentiation was usually retained by the tumour implants.

Two tumours failed to grow in control animals. Examinations of implants from one, a mesenchymal sarcoma, revealed viable tumour cells, but implants of the other, a colonic carcinoma, contained only a few cells of possible tumour origin.

The effect of nitrogen mustard was studied on implants of 13 tumours (Table III). Five showed measurable growth during the period of cytotoxic therapy. On histological examination 2 of these were assessed as viable and 3 as non-viable.

TABLE III.—*The Response to Nitrogen Mustard of Cheek-pouch Grafts of Human Tumours*

Eight implants did not grow. Two contained viable tumour cells (Fig. 5), but the remaining 6 were not viable (Fig. 6).

The effect of methotrexate was also studied on implants from 13 tumours (Table IV). Three showed measurable growth during the period of cytotoxic therapy. On histological examination 2 of these were assessed as viable and one

TABLE IV.—*The Response to Methotrexate of Cheek-pouch Grafts of Human Tumours*

as non-viable. Ten implants did not grow. One contained viable tumour cells but the remaining 9 were not viable.

On the basis of these observations 6 tumours were assessed as sensitive to the action of nitrogen mustard. These were tumours which did not grow and were found to be non-viable after therapy. In one case, however, this finding was invalidated because 2 control implants also failed to grow. Two tumours which grew during treatment and remained viable were judged to be resistant. The results with the remaining 5 tumours were equivocal.

Nine tumours were assessed as sensitive to the action of methotrexate. In 2 of these cases this finding was invalidated because control implants also failed to grow. Two tumours which grew during treatment and remained viable were judged to be resistant. The results with the remaining 2 tumours were equivocal.

### DISCUSSION

The idea of using an *in vivo* technique to predict the clinical efficacy of cytotoxic agents is attractive in theory. But before a technique sufficiently reliable to be of practical value can be established, the problems of obtaining growth of heterologous tumours, and then of assessing the effects of cytotoxic drugs on these growing tumours have to be overcome.

The present studies confirm that many human tumours will grow for short periods at the hamster cheek pouch. They also demonstrate that the Sanders' observation chamber can be conveniently used to measure the growth and response to cytotoxic drugs of these tumours *in vivo*. The finding that the microvascular response induced by grafts of individual human tumours, and the growth and viability of these implants, is very similar in different hamsters suggests that it should be possible to obtain consistent and reproducible results from studies with cytotoxic drugs.

If the results of cytotoxic studies on human tumour grafts growing in the hamster are to have a practical, clinical application, the situation in the animal host must approximate as closely as possible to that which obtains clinically. In particular the agents being tested should be carried to and from the grafted cells naturally in the blood-stream, as usually occurs in the patient, and should reach these cells in concentrations similar to those attained in the clinical state.

Microscopical studies *in vivo* have demonstrated that cheek pouch implants of cellular human tumours regularly obtain a blood supply within a week of transplantation. Host capillaries grow in and rapidly form an extensive vascular network within the graft. Goodall, Sanders and Shubik (1965), using hamster tumours, have reported that these vessels form a pattern characteristic for each tumour type. Kligerman and Henel (1961) have noted that new tumour vessels in a transplanted murine tumour responded normally to stimulation with drugs such as adrenalin and acetylcholine. These findings suggest that the blood vessels within a tumour graft, although of alien origin, are anatomically and functionally normal, and essentially similar to the tumour vessels in the natural host. This adds support to the view that, provided the drug is blood-borne in both situations, there is no reason why the effect of a cytotoxic agent on a cheek pouch tumour graft should not be the same as the clinical effect of the drug upon the same tumour.

The problem of ensuring that cytotoxic agents reach implanted tumour cells in concentrations identical with those obtained clinically has not been completely solved. Pinkel (1958) and Freireich *et al.* (1966) have claimed that the most accurate method of obtaining comparable anti-tumour effects from cytotoxic drugs in different species is to use dosages based upon an estimate of body surface area. However, at best this is likely to produce drug activity which is only approximately equivalent. A more precise method of correlating dosages of cytotoxic agents in man and in the hamster will have to await a detailed examination and comparison of the response of human tumours tó drugs in the 2 species.

All human neoplasms inevitably regress within a few weeks of transplantation to the hamster cheek pouch. Therefore, the time during which the effects of cytotoxic drugs can be studied is limited. Any changes in the size of the grafts during this relatively short period are likely to be small. For this reason an accurate method of measuring the growth of tumour implants at a microscopic level is essential if useful results are to be obtained.

The Sanders' observation chamber fulfils this need. Comparative measurements *in vivo* and in stained microscopical sections have shown that the edge of a grafted tumour can be delineated with sufficient accuracy within the chamber to allow changes in graft size to be assessed. But although the growth of tumour grafts can be measured by this means, it is not possible to recognise whether the cells within an implant are dead or alive by observation *in vivo*. This can only be determined by histological examination of the tumour after its excision from the hamster. Therefore, a combination of measurement of tumour growth *in vivo* and subsequent examination of the fixed and stained implant is necessary for a full evaluation of the effects of cytotoxic drugs on tumour grafts.

In the present studies comparison of the results obtained in animals treated with cytotoxic drugs and in untreated controls has enabled human tumours to be graded with regard to their sensitivity to nitrogen mustard and methotrexate. Some of the tumours were found to be sensitive or resistant to the drugs, but in a substantial proportion of cases the results were equivocal. In these instances the tumours either grew during the period of cytotoxic therapy and were then found to be non-viable at the end of treatment, or else they failed to grow but remained viable. The significance of these findings is not certain, but possibly in the first instance the explanation is that although the tumour implants showed some growth during cytotoxic therapy, the cumulative effects of the drug resulted in destruction of tumour cells by the end of treatment. This suggests that, although some early growth was possible, these tumours were essentially responsive to the drugs. In the second instance the explanation would appear to be that the cytotoxic agents, although effective in preventing cell-division, and hence tumour growth, did not cause cytolysis. This too suggests that the tumours were responsive to some extent.

The results of these experiments indicate that human tumours transplanted to the hamster cheek pouch show demonstrably different responses to cytotoxic drugs administered in identical doses and for identical periods of time. This suggests that tumours growing at the cheek pouch are able to retain their individual sensitivities to cytotoxic agents, and that in consequence it may be possible eventually to use the cheek pouch technique to predict the very variable clinical sensitivities of human tumours to cytotoxic drugs. However, although one might be tempted to infer that the response of a human tumour to a cytotoxic agent clinically is likely to be similar to its response to the same drug in the hamster, in the final analysis this can only be established by a careful examination and direct comparison of the eslults obtained with individual tumours in the hamster and in man.

SUMMARY

The Sanders' observation chamber has been used to examine the blood supply, growth and response to cytoxic drugs of human tumours transplanted to the hamster cheek pouch. Grafts from different tumours varied widely in their vascularisation and growth but grafts from the same tumour showed a consistently similar microvascular and growth pattern in different hamsters. Observations on the effect of nitrogen mustard and methotrexate on tumour grafts suggest that human tumours retain their individual sensitivities to cytotoxic agents while growing at the cheek pouch, and that it may therefore be possible to use the technique to predict the clinical response of human tumours to cytotoxic drugs.

This work was carried out in the Surgical Unit at the Westminster Hospital under the direction of Professor Harold Ellis to whom I am much indebted for encouragement and advice. Thanks are also due to Mr. J. A. Haynes and Mr. P. Moore for technical assistance and to Miss P. T. Brock for preparing the histological sections. The study was completely supported by a grant from the British Empire Cancer Campaign for Research.

### REFERENCES

Billingham, R. E., Ferrigan, L. W. and Silvers, W. K.—(1960) *Science, N.Y.*, **132**, 1488.

Burt, F. B., Pavone-Macaluso, M., Horns, J. W. and Kaufman, J. J.—(1966) *J. Urol.*, **95**, 1.

Cohen, S. N.—(1961) *Proc. Soc. exp. Biol. Med.*, **106**, 677.

Delorme, E. J., Goodwin, C. M., Gowing, N. F. C., Moreman, K. G. and Wylie, J. A. H.—(1965) *Br. J. exp. Path.*, **46**, 530.

Ellis, H. and Sewell, I. A.—(1966) *Br. J. Surg.*, **53**, 153.

Freireich, E. J., Gehan, E. A., Rall, D. P., Schmidt, L. H. and Skipper, H. E.—(1966) *Cancer Chemother. Rep.*, **50**, 219.

Goodall, C. M., Sanders, A. G. and Shubik, P.—(1965) *J. natn. Cancer Inst.*, **35**, 497.

Handler, A. H.—(1958) *Ann. N.Y. Acad. Sci.*, **76**, 775.

Handler, A. H., Davis, S. and Sommers, S. C.—(1956) *Cancer Res.*, **16**, 32.

Hertz, R.—(1959) *Proc. Soc. exp. Biol. Med.*, **102**, 77.

Kligerman, M. M. and Henel, D. K.—(1961) *Radiology*, **76**, 810.

Korngold, L. and Lipari, R.—(1955) *Cancer Res.*, **15**, 159.

Levan, A.—(1956) *Cancer, N.Y.*, **9**, 648.

Patterson, W. B., Patterson, H. R. and Chute, R. N.—(1957) *Cancer, N.Y.*, **10**, 1281.

Pierce, B., Dixon, F. J. and Verney, E. (1958) *Cancer Res.*, **18**, 204.

Pinkel, D.—(1958) *Cancer Res.*, **18**, 853.

Sanders, A. G.—(1963) *J. Anat.*, **97**, 631.

Sewell, I. A.—(1966) *J. Anat.*, **100**, 839.

Sommers, S. C., Friedell, G. H. and Robinson, C. R.—(1966) *Cancer, N.Y.*, **19**, 674.

Toolan, H. W.—(1955) *Trans. N.Y. Acad. Sci.*, **17**, 589.

Warren, B. A. and Shubik, P.—(1966) *Lab. Invest.*, **15**, 464.

Yohn, D. S., Hammon, W. M., Atchison, R. W. and Casto, B. C. (1962) *Cancer Res.*, **22**, 443.